# Temporal and spatial variability of terrestrial diatoms at the catchment scale: controls on communities

**DOI:** 10.7717/peerj.8296

**Published:** 2020-01-03

**Authors:** Jasper Foets, Carlos E. Wetzel, Adriaan J. Teuling, Laurent Pfister

**Affiliations:** 1 Environmental Research and Innovation Department, Luxembourg Institute of Science and Technology, Belvaux, Luxembourg; 2 Department of Environmental Sciences, Wageningen University and Research, Wageningen, Netherlands; 3 Faculty of Science, Technology and Communication, University of Luxembourg, Belval, Luxembourg

**Keywords:** Ecology, Soil, Algae, Indicator species, Agriculture

## Abstract

Diatoms are generally regarded as inhabitants of water bodies. However, numerous taxa are able to survive and reproduce in a variety of non-aquatic ecosystems. Although terrestrial diatoms are discussed extensively in the literature, most of those studies covered floristic aspects and few information exists on their ecology. This lack of knowledge thwarts their potential use as environmental markers in various applications. As a way forward, we investigated the seasonal patterns and the role of different disturbances on the community composition. We collected soil diatom samples in 16 sites across the Attert River basin (Luxembourg) every 4 weeks for a period of 14 months. Our results indicate that forests create a stable microhabitat for diatoms and that temporal variation of the diatom communities is mainly controlled by farming practices rather than seasonal changes in environmental variables. We also found out that communities need one to 2 months to reestablish a new, stable community after a significant change in the environment. We were able to confirm the applicability of the Pollution-Sensitivity Index (IPS) to identify anthropic disturbances.

## Introduction

Diatoms are generally regarded as inhabitants of water bodies. However, numerous taxa are able to survive and reproduce in a variety of terrestrial ecosystems such as soils, mosses, wet walls and rocks (Smol & Stoermer, 2010). Generally, those environments are much harsher for diatoms than aquatic habitats (Ress, 2012). Variables such as moisture and temperature could vary significantly over the course of a day or between two consecutive days and hence, diatoms could experience frequent and prolonged periods of desiccation in certain moments. Therefore, terrestrial diatom species have developed several methods (both morphologically and physiologically) to cope with the variable and moisture limited conditions. For instance, as the characteristic siliceous cell wall consists of many pores, they often decrease its number or create structures to enclose them on the out- or inside to prevent water loss (Lowe et al., 2007; Ress, 2012). This adaptability suggests that diatoms not only survive on soils, but also could thrive and even be the dominant algal group.

Freshwater diatoms are very well studied and generally regarded as a good bio-indicator for water quality assessment due to their high diversity, rapid turn-over and sensitivity to numerous environmental conditions (e.g., pH, organic and inorganic pollution) (Prygiel & Coste, 1993; Van Dam, Mertens & Sinkeldam, 1994). In order to estimate degradation levels of water bodies several diatom-based indices such as Biological Diatom Index (BDI; (Lenoir & Coste, 1996)), Pollution-Sensitivity Index (IPS; (Cemagref, 1982)) and the Eutrophication/Pollution Index-Diatom based (EPI-D; (Dell’uomo & Torrisi, 2011)) have been developed and incorporated in legislatives. While the ecology of aquatic species is better understood, little ecological information exists on terrestrial diatoms, despite being discussed in an extensive literature. This is because most of the publications consist solely of floristic lists (i.e., qualitative data) and researchers often used culturing methods prior to microscope examination. Due to the latter, many diatom species were overlooked and relative abundances were not reflecting the prevailing environmental conditions (Broady, 1979). Nevertheless, some notable ecological studies exist. Van de Vijver, Ledeganck & Beyens (2002) found that moisture content and nutrient concentrations (particularly S0_4_^−2^ and P0_4_^−3^) were the main environmental variables influencing the diatom communities. Whereas in the study of Antonelli et al. (2017) pH and anthropic disturbances caused by farming practices were seen as the principal explanatory variables. Similarly, studies comparing different disturbance factors showed that soil diatoms are quite responsive to agricultural practices (Heger, Straub & Mitchell, 2012; Stanek-Tarkowska et al., 2018; Stanek-Tarkowska & Noga, 2012). It was even possible to separate those different land use types based on IPS values (Antonelli et al., 2017). Although knowing that anthropic disturbances play a key role in shaping the species composition of soil diatom communities, many details on species occurrences and sensitivities are still unknown.

The variability of physical, chemical and biological conditions over the course of a year cause significant seasonal changes in the biomass and composition of dominant freshwater diatom species in temperate regions (Wetzel, 2001). For example, different diatom species reach peak populations at different times during the annual cycle, thereby growing under different environmental conditions (Köster & Pienitz, 2006). This is, however, not always the case in (very) stable ecosystem types such as springs where seasonality is often totally absent (Cantonati, Gerecke & Bertuzzi, 2006). While this behavior is very well documented for aquatic diatoms, it is still an open question in soil diatom ecology. To the best of our knowledge, only two studies briefly discussed this topic. However, both had contrasting results with Lund (1945), who observed a garden soil for several years, reporting no seasonality in the community composition, whereas Antonelli et al. (2017) found signs of seasonality reflected by the different species compositions in the communities over three seasons. It should be noted that in both cases the temporal resolution was relatively coarse. Lund (1945) took his samples when environmental conditions were “optimal” leading to sampling 26 of the possible 58 months. Antonelli et al. (2017), on the other hand, sampled once every 4 months. In order to have a better idea of the behavior of terrestrial diatoms and subsequently to use them in future applications (e.g., soil bio-indicator, hydrological tracer), it is essential to understand the seasonal patterns of the communities and their primary causes.

Natural catchments are very heterogeneous areas and exhibit spatio-temporal variability of soil moisture, land surface temperature and vegetation at a range of scales. (He et al., 2019; Troch et al., 2009; Western et al., 1999). Within a catchment those variables are linked through the distribution of energy and temperature (varying with exposure and altitude), and with varying wetness conditions (varying from infiltration and deep groundwater tables in the upslope parts to exfiltration and shallow groundwater tables in valleys and convergence zones) (Burt & Butcher, 1985). Nevertheless, since most of the variability in hydrological conditions and radiation/temperature will by definition occur at the catchment scale, this has long been a central scale in (eco)hydrological research. One such heterogeneous catchment is the Attert River basin in Luxembourg, which is characterized by a wide variety in physiographic settings (Pfister et al., 2017). As a result, it has been the centerpiece for recent studies on terrestrial diatoms and particularly their use as hydrological tracers (Pfister et al., 2017). In the light of those studies, many different areas of the basin were investigated and soil diatom communities characterized. Klaus et al. (2015) examined 302 samples (including stream samples) in six nested catchments and found 85 different terrestrial species. Also, Antonelli et al. (2017) took soil diatom samples at 34 locations across the whole basin, while Barragán, Wetzel & Ector (2018) sampled around four of those to test a new sampling strategy. As a result of those studies, several new species were discovered and described (Barragán, Ector & Wetzel, 2017; Wetzel et al., 2014, 2013). In spite of previous efforts, a full, detailed characterization of terrestrial diatoms over the catchment and across seasons is lacking.

The purpose of our study is to provide more detailed information on the temporal and spatial variability of soil diatoms. This paper mainly focuses on the temporal variability of terrestrial diatom communities and has special attention for seasonal patterns and the role of land uses (e.g., forest, agricultural fields and (un)disturbed grasslands), as anthropic disturbances, on the community composition.

## Materials and Methods

### Study area and weather conditions

**Figure 1:**
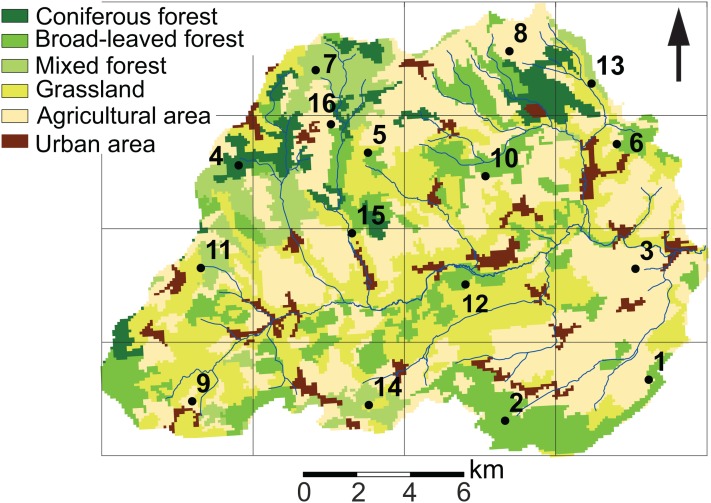
Land use characteristics of the Attert River basin in Luxembourg and Belgium. The map shows the 5 × 5 km grid and the 16 sampling locations. Source: Modified from Corine Land Cover (2018).

Diatom sampling was conducted in the Attert River basin, which covers an area of approximately 249 km^2^ and is located for the most part in Luxembourg with the exception of the western part, which is located in Belgium (49°46′13.0″ N, 5°59′9.2″ E). The topography in the north of the basin is typified by a sub-horizontal plateau with elevations ranging between 450 m and 500 m and steep slopes, whereas the southern part has gently sloping lowlands at 300 m a.s.l. (Pfister et al., 2015). Moreover, the area has a complex geological setting. Keuper sandy marls and Jurassic sandstone dominate the southern part of the basin, while the northern part consists of Devonian schists and Luxembourg red sandstone (“Buntsandstein”). Recent alluvial depositions are present in the vicinity of streams. Soil types generally follow the bedrock geology. Cambisols dominate the schists area, whereas Stagnosols and Planosols are the dominant types on marls. Leptosols, Arenosols and Podzols characterize the Luxembourg red sandstone (Cammeraat et al., 2018). Land cover in the basin consists of farmland, grasslands and forested areas (Fig. 1). Farmland is mainly used for growing corn, wheat and rapeseed, while grasslands are predominantly used for cattle grazing. European beech (*Fagus sylvatica* L.) and spruce (*Picea abies* L.) dominate the forested areas. The climate of the basin is semi-oceanic.

The sampling campaign was performed monthly from October 2017 to November 2018. The summer prior to sampling was relatively wet (on average 88 mm/month), whereas the average monthly precipitation during the sampling period was 56.4 ± 33 mm. The maximum monthly precipitation was observed in December 2017 and January 2018 (respectively 111.9 mm and 123.5 mm). Monthly precipitation reached a minimum in October 2018 (18 mm). July and August were the warmest months, reaching average temperatures of 20.5 °C and 18.7 °C, while most freezing days were encountered during February and March (28 and 12) (Data retrieved from Administration des Services Techniques de l’Agriculture (ASTA)). Overall, 2018 was characterized by a cold winter and an exceptionally dry and warm summer period.

### Sample collection, preparation and diatom identification

In order to capture the intra-annual and spatial variability of diatom communities, soil diatom samples were taken at the soil surface in 16 sites around the catchment every 3–5 weeks for a period of 14 months (*n* = 224 samples). In order to systematically cover the whole area, a grid of 5 × 5 km was mapped on the study area (Fig. 1). Next, at least one fixed sampling point per cell was chosen considering the differences in geology, soil and land use features, and previous diatom research in the area (Antonelli et al., 2017; Barragán, Wetzel & Ector, 2018). At each sampling campaign, a description of the site was made in order to keep track of the environmental changes in between the sampling periods. Characteristics such as type of land use, disturbances, height of the lower vegetation (i.e., indicator for mowing/grazing) were noted. Also, one sample per site was taken for diatom analysis. According to Barragán, Wetzel & Ector (2018), this should be sufficient to have a diatom community representative for a homogeneous area of at least 75 m^2^.

We used metal rings (diameter: 5.6 cm) to collect small soil cores. Upon arrival in the lab, diatoms were extracted by rinsing the soil surface with MQ water until a 50 mL falcon tube was filled (see Barragán, Wetzel & Ector, 2018). The samples, consisting of soil particles and water, were then fixed with 70% ethanol. Diatom slides were prepared for microscopic counts following the CEN 13946 procedure (European Committee for Standardization, 2003). A minimum of 200 valves were counted and identified on each slide along random transects using a Leica DMR light microscope with a × 100 oil immersion objective and a magnification of × 1,000 (Bate & Newall, 2002).

Diatom identifications were based on following taxonomic references: Ettl & Gärtner (2014); Hustedt (1941); Krammer (2000); Lange-Bertalot (2001); Lange-Bertalot et al. (2017); Levkov, Metzeltin & Pavlov (2013); Wetzel et al. (2015, 2013).

For scanning electron microscope (SEM) analysis, parts of the oxidized suspensions were filtered and rinsed with additional deionized water through a three µm Isopore^™^ polycarbonate membrane filter (Merck Millipore). Filters were mounted on aluminum stubs and coated with platinum (30 nm) using a BAL-TEC MED 020 Modular High Vacuum Coating System for 30 s at 100 mA. A Hitachi SU-70 ultra-high-resolution analytical field emission SEM (Hitachi High-Technologies Corporation, Tokyo, Japan), operated at 5 kV and with a 10 mm working distance, was used for the analysis. SEM images were taken using the upper (SE-U) detector signal. Photomicrographs were digitally manipulated and plates containing light and scanning electron micrographs were created using CorelDraw X8. For a more detailed and complete description of the methodology, we refer to Barragán, Wetzel & Ector (2018).

### Statistical analysis

Before carrying out the statistical analysis, the species dataset was reduced by only keeping the taxa with a relative abundance of at least 2% in at least two samples or 4% in one sample. Samples which contained less than 100 valves were removed from the analysis (18 of the 224 samples). Of the 18 samples, 12 were taken during the dry summer period and in the aftermath of it (July: 1, August: 3, September: 5, October: 3). A Detrended Correspondence Analysis (DCA) was performed to see how communities relate to each other and to estimate the length of the gradient (Hill & Gauch, 1980). The analysis revealed a gradient longer than 5.0 SD (Standard deviation of species turnover). Also, the non-parametric statistical test Analysis of Similarities (ANOSIM) using Bray–Curtis as a dissimilarity metric, was used to test if communities significantly differ between land use types (Clarke, 1993). Temporal variation was assessed using the same analysis performed for each type of land use. Significance of both analyses were tested with the default Monte Carlo permutation test (perm-999). The effect of geographical distance on the species composition of the communities was assessed with a Mantel test. Species data were transformed (Bray–Curtis) to a dissimilarity matrix and tested if there was significant correlation with the geographical distance matrix. The latter was obtained using the *rdist.earth* function from the R-package *fields* (Nychka et al., 2017).

The characteristic species and their indicator value for the different land uses (i.e., agricultural field, grazed grassland, agricultural grassland, undisturbed grassland and forest) were assessed using the option *IndVal.g*, which is implemented in the *multipatt* function included in the *indicspecies* package. This function takes into account species presences/absences and relative abundances per type of land use (De Cáceres, Legendre & Moretti, 2010). Also, the Shannon–Wiener index was computed for each sample with the function *diversity*. Additionally, IPS values were calculated (Cemagref, 1982) for the five different land uses to check if differences in communities could be expressed by a single value. This index takes into account the abundance of each species in the sample, their sensitivity value (i.e., sensitivity to pollution) and their indicator value (i.e., relative probability of each species to occur in one of five saprobity classes). Calculations for both, Shannon–Wiener and IPS index, were done on the whole dataset of soil diatom species. The IPS indices were computed with the software Omnidia (version 6.0.8, 2018) (Lecointe, Coste & Prygiel, 1993) and compared with ANOVA (analysis of variance). IPS was chosen for this study, since it is the index with the higher number of taxa included and the most updated. Furthermore, a CONISS cluster analysis was done for sites 9, 10 and 13 to see when diatom community composition changed following land management practices. This was conducted with the function *strat.plot* in the *Rioja* package (Juggins, 2017). All aforementioned statistical analyses were performed using the R statistical program (R v. 3.5.0.; http://www.r-project.org/) and additional functions from the R-package *vegan* version 2.5–4 (if not mentioned differently).

## Results

We identified a total of 302 different taxa, including varieties, subspecies and forms, belonging to 44 genera. After using the cutoff criteria, 111 species (90% of the total valves counted) were kept in the statistical analyses. Most species rich genera were *Pinnularia*, *Nitzschia*, *Mayamaea*, *Stauroneis* and *Sellaphora* comprising respectively 17, 14, 12, 6 and 5 taxa. Whereas *Hantzschia amphioxys* (Ehrenberg) (19% of the total valves counted), *Mayamaea permitis* (Hustedt) Bruder & Medlin (8%), *Nitzschia pusilla* (Kützing) Grunow emend. Lange-Bertalot (6%), *Pinnularia obscura* Krasske (5%) and *Hantzschia abundans* Lange-bertalot (5%) were the most abundant species. An average species richness/sample of 22 ± 8.3 was observed with the lowest values in December (18.3 ± 6.3) and October 2018 (17.8 ± 7.1). Maxima were noted in November 2017 (49), February (45) and July (45). The Shannon–Wiener index was similar between the 14 months (average 2.01 ± 0.17).

**Figure 2:**
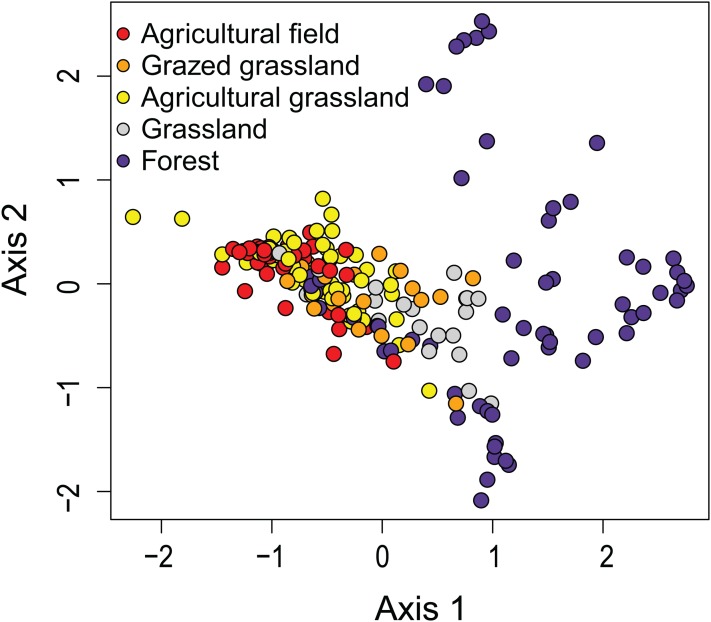
Results of the Detrended Correspondence Analysis (DCA). In DCA, samples are ordered based on community (dis)similarities. Samples close to each other suggest similar communities. Samples are colored according to their land use type.

**Figure 3:**
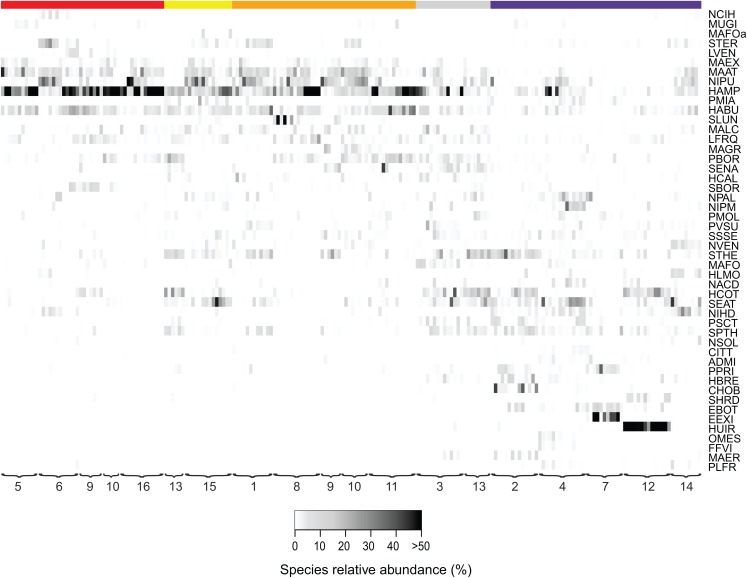
Relative abundances of terrestrial diatom species over the sampling period. Only diatom taxa with the most significant indicator value are listed. Diatom taxa are abbreviated following Omnidia. All codes and respective full species names could be found in the (Table S1). Sites (1–16) are ordered according to their land use type (red, agricultural field; yellow, grazed grassland; orange, agricultural grassland; gray, grassland; purple, forest) and samples are ordered chronologically. The color indicates the taxon relative abundance (white, absent; black, >50%).

The first two axes of the DCA explained respectively 9.0 and 6.6% (both *P* < 0.05) of the total variance. The DCA clustered sites regarded as agricultural field, grazed grassland and agricultural grassland together, while (undisturbed) grassland is set apart from that cluster (Fig. 2). Samples taken in forested areas are spread along the second axis of the plot. Additionally, we were able to distinguish four different sites (12, 7, 4, 2) within those samples, suggesting that species composition of the communities is quite different from each other and that the composition generally does not change much over time (Fig. 3). Indeed, site 12, a deciduous forest located on marls, had repeatedly very high abundances of *Humidophila irata* (Krasske) Lowe, Kociolek, J.R. Johansen, Van de Vijver, Lange-Bertalot & Kopalová and to a lesser extent *Sellaphora harderi* (Hustedt) J. Foets & C.E. Wetzel, while species of the genus *Eunotia* together with *Pinnularia perirrorata* Krammer and *Pinnularia schoenfelderii* Krammer (not on Fig. 3) were often very abundant on site 7. It is also one of the few sites where *H. amphioxys* was totally absent during the whole sampling period. Site 7 is situated in the schists area and the soils are typically very acidic, since the area is dominated by pine trees. On site 4, located in the schists area and covered by mixed forest, communities are mainly influenced by the riparian zone of the stream nearby. Hence, communities are generally very diverse, in particular with many different *Nitzschia* and *Navicula* species present in low abundances. Finally, site 2, characterized by sandy soils and a mixed forest, is generally dominated by *Chamaepinnularia obsoleta* (Hustedt) C.E. Wetzel & Ector, *Stauroneis thermicola* (J.B. Petersen) J.W.G. Lund and *Diadesmis contenta* var. *biceps* (Grunow) P.B. Hamilton.

**Figure 4:**
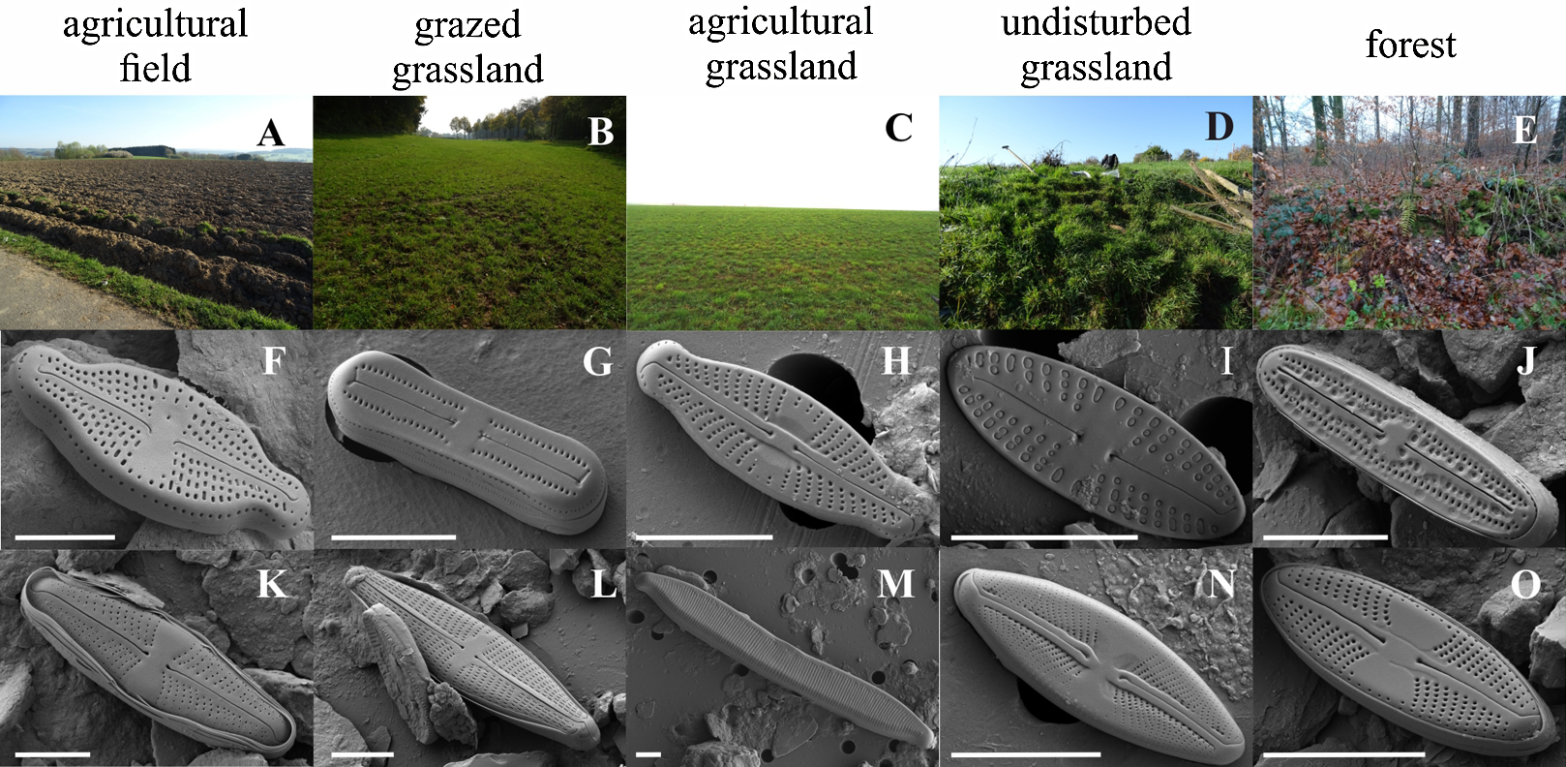
Habitat impression and scanning electron images of some of the dominant diatom species. Habitats: A, agricultural field (site 9); B, grazed grassland (site 15); C, agricultural grassland (site 9); D, undisturbed grassland (site 13); E, forest (site 12). Diatom taxa: F, *Luticola ventricosa* (Kützing) D.G. Mann in Round, Crawford & D.G. Mann; K, *Stauroneis* (cf.) *borichii* (J.B. Petersen) J.W.G. Lund; G, *Humidophila contenta* (Grunow) Lowe, Kociolek, J.R. Johansen, Van de Vijver, Lange-Bertalot & Kopalová; L, *Navicula veneta* Kützing; H, *Sellaphora lundii* C.E. Wetzel, Barragán & Ector; M, *Hantzschia calcifuga* Reichardt & Lange-Bertalot; I, *Mayamaea fossalis* (Bock) Lange-Bertalot; N, *Sellaphora nana* (Hustedt) Lange-Bertalot, Cavacini, Tagliaventi & Alfinito; J, *Humidophila irata* (Krasske) Lowe, Kociolek, J.R. Johansen, Van de Vijver, Lange-Bertalot & Kopalová; O, *Sellaphora harderi* (Hustedt) J. Foets & C.E. Wetzel. The scale bar is 5 µm. Photo credit: Jasper Foets and Carlos E. Wetzel.

An overview of the different land use types and their characteristic diatom species, selected by the indicator species analysis, is shown in Fig. 4. Indicative species for agricultural fields are *Luticola ventricosa* (Kützing) D.G. Mann in Round, Crawford & D.G. Mann, *Stauroneis* (cf.) *borichii* (J.B. Petersen) J.W.G. Lund and *Navicula cincta* (Ehrenberg) Ralfs var. *heufleri* Grunow in Van Heurck f. *curta*, while *Pinnularia microstauron* (Ehrenberg) Cleve var. *angusta* Krammer, *Navicula veneta* Kützing and *Sellaphora lundii* C.E. Wetzel, Barragán & Ector are characteristic for grazed grasslands. Moreover, *S. lundii* together with *Mayamaea agrestis* (Hustedt) Lange-Bertalot and *Pinnularia subrupestris* Krammer could typically be found on agricultural grasslands, whereas *Mayamaea fossalis* (Krasske) Lange-Bertalot and *Sellaphora nana* (Hustedt) Lange-Bertalot, Cavacini, Tagliaventi & Alfinito are indicative for undisturbed grassland soils. Finally, *H. irata*, *C. obsoleta* and *S. harderi* were only present on forested soils. A complete list with all the characteristic taxa per habitat type is given in Table S1.

**Figure 5:**
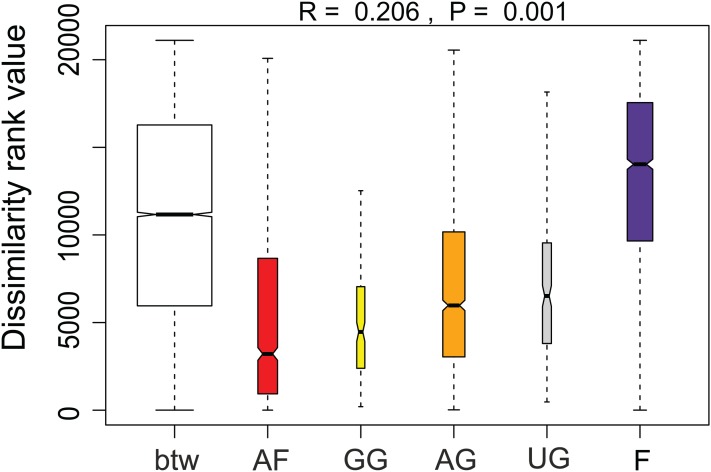
Results of the analysis of similarities. The plot shows the dissimilarity between and within different habitat types. Not overlapping notches indicate that the medians significantly differ (Chambers et al., 1983). btw, between-group dissimilarity; AF, agricultural field; GG, grazed grassland; AG, agricultural grassland; UG, undisturbed grassland; F, forest.

**Figure 6:**
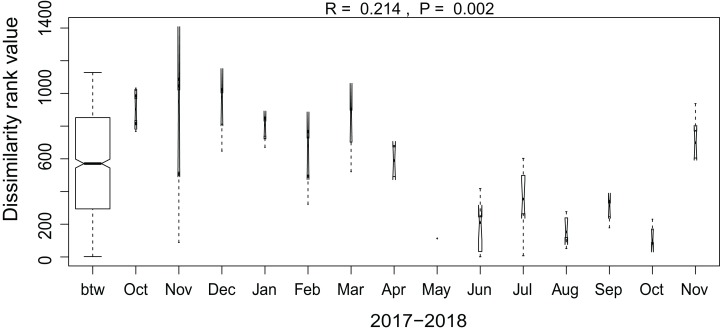
Results of the analysis of similarities. The plot shows the dissimilarity between the different months. Only samples taken on agricultural fields are included in the graph. Not overlapping notches indicate that the medians significantly differ (Chambers et al., 1983). btw, between-group dissimilarity.

Additionally, we examined which factors were responsible for the (dis)similarities between the different communities over the sampling period. Here, we analyzed whether those differences are rather related to the geographical distances between the sites (communities closer to each other are more similar), the seasonal variation in environmental factors or to external forces (e.g., farming practices), often related to the type of habitat. The Mantel test revealed no significant relations between the geographical distance and the community similarities (*r* = −0.09, *P* = 0.758). Regarding the temporal variation, only communities related to agricultural fields differed significantly between some months (Fig. 5). Nevertheless, we were able to split the samples into three main groups based on the overlap of the notches. The first group contains the period from October 2017 to March 2018 with *Mayamaea atomus* (Kützing) Lange-Bertalot and *N. pusilla* dominant (see Fig. 3). The second group includes the samples taken from June 2018 to October 2018 when *H. amphioxys* became more dominant. April and November 2018 form the third “transition” group with intermediate communities. However, the same analysis was more successful in separating the different land uses (Fig. 6). Only agricultural grassland and grassland could not be separated based on the community compositions, while forested soils have significantly different communities. The latter is clearly visible in Fig. 3, where species in the lower part of the heatmap such as *Sellaphora atomoides* C.E. Wetzel & Van de Vijver, *N. veneta*, *S. thermicola*, *Stauroneis parathermicola* Lange-Bertalot in Hofmann, Werum & Lange-Bertalot and *Humidophila* spp. are more abundant on undisturbed sites. On the contrary, disturbed areas are especially dominated by *H. amphioxys*, *H. abundans*, *N. pusilla* and *M. atomus*. The Shannon–Wiener index also revealed a significant lower diversity for agricultural fields compared to the other types (*F* = 8.8, df = 4, *P* < 0.0001). Thus, the type of habitat has stronger effects on the diatom community than the seasonal and geographical factors.

Also, large shifts in the community composition were observed following land management practices. An example is site 9. From October to March the site was regarded as an agricultural grassland and except for mowing once or twice, no other disturbances took place (see Fig. 3). During that period, the community consisted mainly of *S. thermicola*, *N. pusilla*, *M. atomus* and *M. permitis*. Between the sampling in March and April, the site was plowed and manured transforming the area to an agricultural field. The field remained in this condition for the rest of the study period in which crops were grown and harvested twice. Following those farming practices, the species composition changed, after a lag-phase of 1–2 months, to a community dominated by *H. amphioxys*, *H. abundans* and *S. borichii*. A more detailed figure of the last example is shown in the (Fig. S1). Furthermore, similar community shifts occurred in sites 10 (from agricultural grassland to agricultural field in June) and 13 (from undisturbed to grazed grassland in June). Also there, *H. amphioxys* and *H. abundans* replaced the more sensitive *N. pusilla*, *M. atomus* and *M. permitis*.

**Figure 7:**
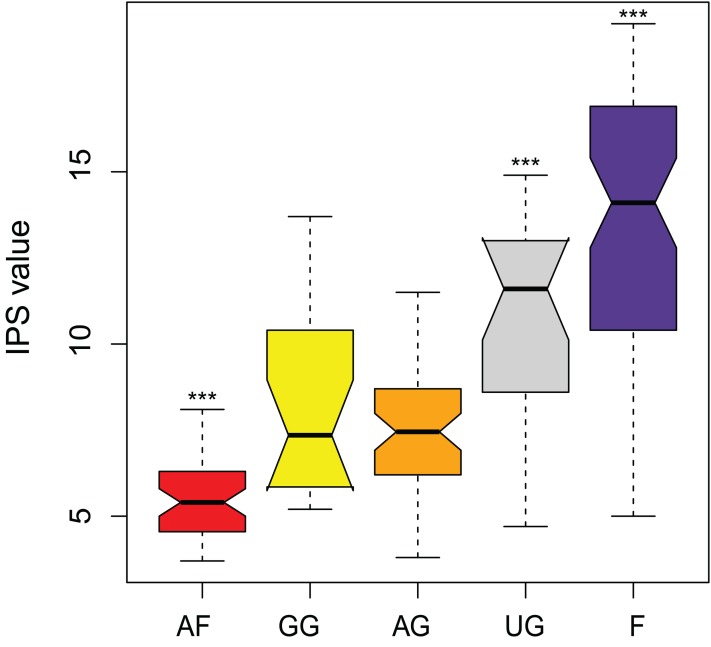
Comparison of pollution-sensitivity index (IPS) values between habitat types. AF, agricultural field; GG, grazed grassland; AG, agricultural grassland; UG, undisturbed grassland; F, forest. Not overlapping notches indicate that the medians differ (Chambers et al., 1983). ***, Habitat is significantly different from all the other habitats.

The IPS values show to be a good parameter in separating the different land uses (*F* = 59.6, df = 4, *P* < 0.0001) (Fig. 7). Agricultural fields, undisturbed grassland and forest could be separated from all the other land use types. Only the samples from the semi-disturbed types, grazed grassland and agricultural grassland could not be separated from each other (*P* = 0.89). Also, agricultural fields comprise the species which are least sensitive to pollution (= lowest values), while undisturbed sites consist mainly of sensitive species (= highest values). Unfortunately, not all of the species have attributed IPS values yet (on average 85.6 ± 5.8%) and thus the final calculations included only 90.7% (± 11.9%) of the total abundance.

## Discussion

### Seasonal variation

Our research showed that seasonal variation in soil diatom communities was rather limited or almost absent when looking at the communities in the forest sites. A reason behind this stationarity could be that forests create a relatively stable microhabitat for diatoms. They exhibit generally a higher and more constant moisture than grasslands (James et al., 2003). The temperature fluctuations are usually smaller and maximum temperatures are lower. Also, the forest canopy provides a good protection against direct UV-radiation, which is considered to be harmful to algae (Ress, 2012), and ensures that the topsoil layer dries out less quickly. Although, forest soils are receiving high amounts of leaf litter and seasonal changes resulting from that (i.e., decomposition and mineralization processes), but this does not seem to have any visible effect on soil diatoms. Similar to forests, several spring types have shown to create a stable environment for aquatic diatoms (i.e., fairly constant pH, temperature and trophy level), leading to limited seasonal changes in the communities (Cantonati, 1998). Cantonati, Gerecke & Bertuzzi (2006) therefore concluded that this actually facilitates the use of diatoms as ecological indicators since fewer numbers of surveys are required. The same principle could be applied to forest soils, meaning that sampling once a year should be sufficient.

In addition to the minor seasonal influences in forests, we did not observe any significant temporal variation in the other habitat types, except for communities living on agricultural fields. There, we observed a general change from *Mayamaea* to *Hantzschia* dominated communities. However, this variation could be explained by farming practices such as plowing and manuring, since the compositional changes in the communities coincides with the beginning (April and May) and ending (October and November) of the “farming season.” Nevertheless, we noticed some small signs of seasonality, especially by some species who are strongly dependent on soil moisture content (e.g., *N. pusilla* (J. Foets, 2018, unpublished data)). Also, the fact that we experienced an exceptionally dry and warm summer period during the sampling campaign, did not help to observe potential seasonality since sufficient moisture availability is essential for diatom survival and reproduction (Van de Vijver, Ledeganck & Beyens, 2002). So, seasonality in diatom community composition could occur in (disturbed) grassland soils, but it is rather unlikely that it will play a significant role in the composition of soil diatom communities.

### Habitat variation

We found that the type of land use was a key factor in defining the species composition. This result is consistent with previous works that showed different communities with different levels of disturbance (Antonelli et al., 2017; Stanek-Tarkowska et al., 2018; Stanek-Tarkowska & Noga, 2012; Vacht et al., 2014). In contrast to these works, we compared two to three habitat types more at the same time. Although our analysis could not split the communities sampled on agricultural grasslands from the ones taken on undisturbed grasslands, our results still show that species are perhaps more sensitive and indicative for anthropic disturbances than we previously thought. Indeed, we were able to define several indicator species for each type of land use and when comparing those species with earlier works, we noticed many similarities. For instance, Barragán, Wetzel & Ector (2018) also reported *S. lundii* from a meadow and *P. perirrorata* and *Eunotia* spp. from a forest site, whereas Stanek-Tarkowska et al. (2013); Stanek-Tarkowska & Noga (2012) observed *S. borichii* in high abundances on farmland. This habitat specificity of soil diatoms is an interesting outcome and should be explored more often in the future, certainly in the light of using soil diatoms as environmental markers.

Another interesting outcome of this study was the follow-up of the communities after a drastic change in the landscape following land management practices. Two of our 16 sites (9 and 10) went from an agricultural grassland to an agricultural field and one site (13) was transformed from an undisturbed to a grazed grassland. Generally, species colonizing the early stages of a secondary succession have fast immigration rates rather than high reproduction and growth rates (Stevenson, 1986; Stevenson et al., 1991). This is also what we observed in the three cases with *Pinnularia borealis* Ehrenberg, *H. amphioxys* and *H. abundans* often replacing *N. pusilla* and *Mayamaea* spp. The former three taxa are very common, large terrestrial diatoms (±75 µm) and are known to be well-adapted to living in extreme environments (Dodd & Stoermer, 1962; Pipe & Cullimore, 1984; Stock et al., 2018; Van Dam, Mertens & Sinkeldam, 1994). These diatoms are considered to be slow growers, since they spend a large part of their energy in developing complex structures to withstand (very) harsh conditions (Pinseel et al., 2019; Ress, 2012). For example, *Hantzschia* spp. create internal valves to better retain moisture and decrease water loss (Dodd & Stoermer, 1962; Round, Crawford & Mann, 1990). On the other hand, *N. pusilla* and *Mayamaea* spp. are rather small (10–15 µm) and more fragile species (i.e., less silicified cells). We therefore expect them to grow and reproduce faster, but they need preferably a more stabilized habitat. Consequently, changes in land use could have large, similar effects on the community, even in (semi-) disturbed areas.

Besides the identification of important pioneer species, we also noticed that soil diatom communities need between 1 and 2 months to colonize a new, disturbed habitat depending on the prevailing environmental conditions. This recolonization period is longer compared to freshwater communities. They generally need 14 days to 1 month to establish a new, stable community (Blinn, Fredericksen & Korte, 1980; Oemke & Burton, 1986). We stipulate that this difference is mainly due to three reasons: (1) the absolute abundance of soil diatoms is much lower compared to freshwater diatoms (Loescher, 1972) (2) water is a more efficient dispersal mechanism than wind (Kristiansen, 1996) and (3) nutrient exchange via osmosis and reproduction is easier in aquatic conditions. However, it is to be noted that some shifts happened during exceptionally warm, dry weather conditions and therefore recolonization could be shorter in more optimal (moist) conditions. Unfortunately, we were not able to monitor a second succession stage to see if communities go back to their previous composition or to a different one. So, the reestablishment of a stable community on disturbed terrestrial habitats generally happens after 1–2 months with the more tolerant species replacing the rather sensitive ones.

### IPS and future modifications

Despite that the IPS index was developed for water quality assessment, it works very well in separating the different land use types as it is shown here and previously by Antonelli et al. (2017) and Barragán, Wetzel & Ector (2018). Nevertheless, this index should be adapted to terrestrial environments if it is to be used further. In the first place, we should be able now (i.e., with the recent results) to fill in the missing indicator and pollution values for, at least, the most abundant and common terrestrial diatom taxa. The IPS index was chosen, because it is species-specific and is supported by a large and updated database. However, some important terrestrial diatoms are not incorporated in it. For instance, Antonelli et al. (2017) could not include certain abundant species in their analysis, while some of our IPS values were calculated using only 60% of the total valves counted. Secondly, there is a need to update and reevaluate the indicator and sensitivity values, since changes in diatom taxonomy happen very fast leading to new names and/or species descriptions. This in turn leads then to different results in quality indices calculations (Kahlert et al., 2016). Also, both values are inferred from aquatic surveys and many of them are probably not reflecting the behavior of soil diatoms in terrestrial habitats (Antonelli et al., 2017). Finally, we should look at incorporating an additional factor to make the index more specific for terrestrial habitats and eventually decrease the intra-variability and increase the inter-variability between the land use types. For instance, by multiplying that factor it will give more weight to the very specific indicator taxa. Hopefully, with those modifications we will be able to predict anthropic disturbances more precisely in the future.

## Conclusions

In this study, we investigated the temporal and spatial variability of soil diatom communities at the catchment scale. Our results indicate that forests create a stable microhabitat for diatoms and that temporal variation is mainly related to farming practices rather than seasonal changes in environmental variables. This was highlighted by the changes in the species composition of the communities after drastic anthropogenic interventions in the habitat. Our study showed that communities need 1–2 months to reestablish a new, stable community after a significant change in the environment. However, we were not able to observe whether or not communities would go back to their original composition when the habitat would change to its previous state. We eventually confirmed the earlier results of Antonelli et al. (2017) regarding the applicability of the IPS index to predict different anthropic disturbances and stressed that, with the recent results, we should be able to adapt and update the indicator and sensitivity values for terrestrial diatom species so that this will improve the accuracy and predictability of the index.

## Supplemental Information

10.7717/peerj.8296/supp-1 Supplemental Information 1 Temporal succession of the most abundant diatom species of site 9. The site changed from an agricultural grassland to an agricultural field between the sampling in March and April 2018. CONISS cluster analysis indicates two different diatom communities.

10.7717/peerj.8296/supp-2 Supplemental Information 2 List of the significant indicator taxa for the five land use types. A black rectangle indicates that the species is an indicator for that type. AF, agricultural Field; GG, grazed grassland; AG, agricultural grassland; UG, undisturbed grassland; F, forest *, *p*-value < 0.05; **, *p*-value < 0.01.

## Additional Information and Declarations

### Data Availability

The following information was supplied regarding data availability: The data is available at Mendeley: Foets, Jasper (2019), “Temporal and spatial variability of terrestrial diatoms at the catchment scale,” Mendeley Data, v2. DOI 10.17632/9s28gvnr53.2.
