# Supplementary figures and images for: Temporal and spatial variability of terrestrial diatoms at the catchment scale: controls on communities
Source: PeerJ. 2020 Jan 3;8:e8296. doi: 10.7717/peerj.8296 (PMC6944102; doi:10.7717/peerj.8296)

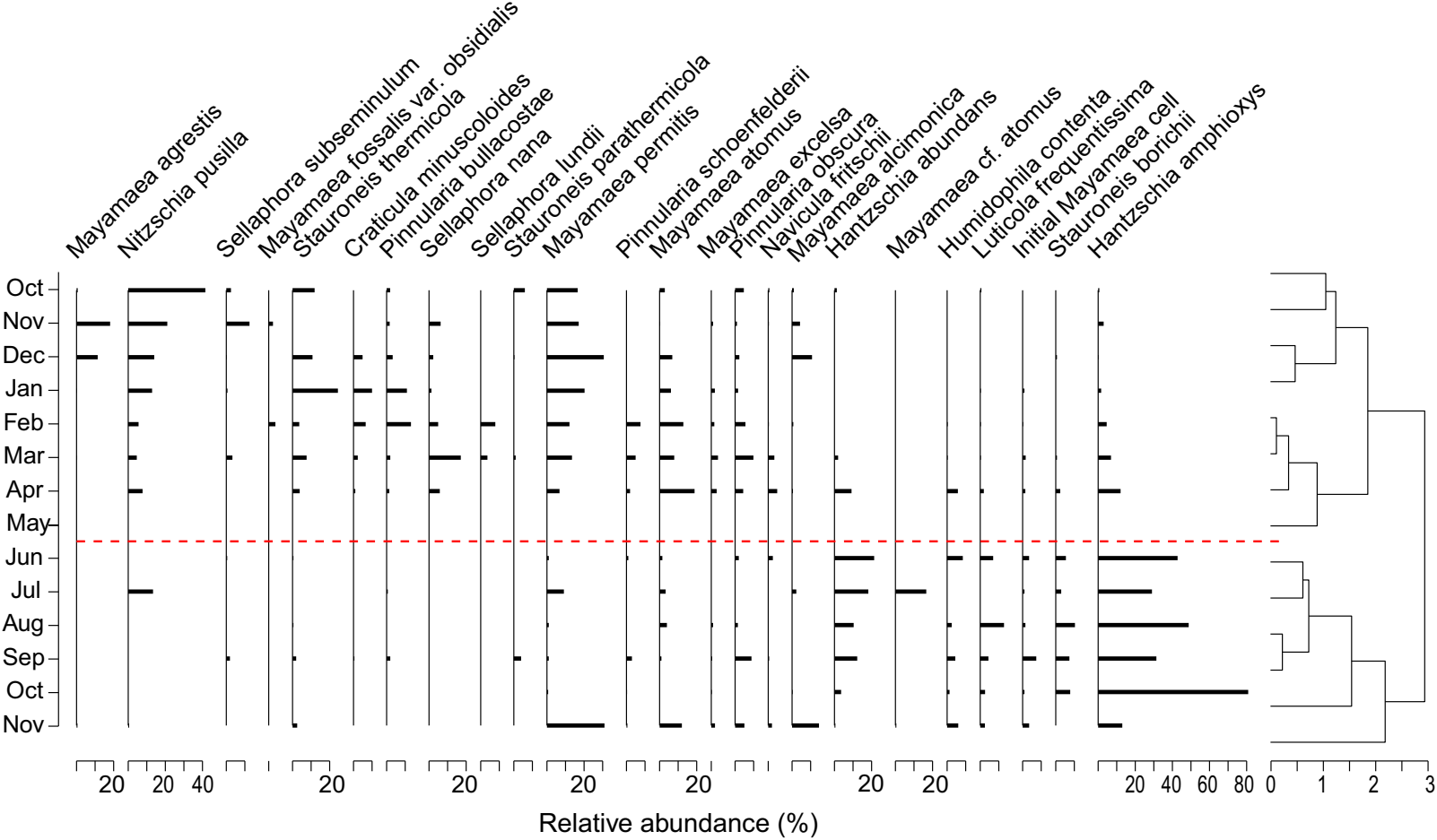

Supplement: Supplemental Information 1 — The site changed from an agricultural grassland to an agricultural field between the sampling in March and April 2018. CONISS cluster analysis indicates two different diatom communities. [file peerj-08-8296-s001.pdf]
