# Supplementary material for: Temporal and spatial variability of terrestrial diatoms at the catchment scale: controls on communities
Source: PeerJ. 2020 Jan 3;8:e8296. doi: 10.7717/peerj.8296 (PMC6944102; doi:10.7717/peerj.8296)
Supplement: Supplemental Information 2 — A black rectangle indicates that the species is an indicator for that type. AF, agricultural Field; GG, grazed grassland; AG, agricultural grassland; UG, undisturbed grassland; F, forest *, p-value < 0.05; **, p-value < 0.01. [file peerj-08-8296-s002.docx]

| Taxon name | CODE | AF | GG | AG | UG | F | P value |
| --- | --- | --- | --- | --- | --- | --- | --- |
| *Achnanthidium minutissimum* (Kützing) Czarnecki | ADMI | 0 | 0 | 0 | 0 | 1 | ** |
| *Cavinula intractata* (Hustedt) Lange-Bertalot | CITT | 0 | 0 | 0 | 0 | 1 | ** |
| *Chamaepinnularia obsoleta* (Hustedt) C.E. Wetzel et Ector | CHOB | 0 | 0 | 0 | 0 | 1 | ** |
| *Eunotia botuliformis* Wild, Nörpel-Schempp et Lange-Bertalot | EBOT | 0 | 0 | 0 | 0 | 1 | ** |
| *Eunotia exigua* (Brebisson) Rabenh. | EEXI | 0 | 0 | 0 | 0 | 1 | ** |
| *Eunotia minor* (Kützing) Grunow | EMIN | 0 | 0 | 0 | 0 | 1 | * |
| *Eunotia palatina* Lange-Bertalot et W. Krüger | EPLT | 0 | 0 | 0 | 0 | 1 | * |
| *Fragilariforma virescens* (Ralfs) Williams et Round | FFVI | 0 | 0 | 0 | 0 | 1 | * |
| *Geissleria paludosa* (Hustedt) Lange-Bertalot et Metzeltin | GPAL | 0 | 0 | 0 | 0 | 1 | * |
| *Halamphora montana* (Krasske) Levkov | HLMO | 0 | 0 | 0 | 0 | 1 | ** |
| *Diadesmis contenta* var. *biceps* (Grunow) P.B. Hamilton | HBIC | 0 | 0 | 0 | 0 | 1 | * |
| *Humidophila irata* (Krasske) Lowe, Kociolek, J.R. Johansen, Van de Vijver, Lange-Bertalot *et* Kopalová | HUIR | 0 | 0 | 0 | 0 | 1 | ** |
| *Meridion circulare* (Greville) C. Agardh | MCIR | 0 | 0 | 0 | 0 | 1 | * |
| *Nitzschia* cf. *palea* (Kützing) W. Sm. | NPALc | 0 | 0 | 0 | 0 | 1 | * |
| *Nitzschia harderi* Hustedt | NIHD | 0 | 0 | 0 | 0 | 1 | ** |
| *Nitzschia solgensis* Cleve-Euler | NSOL | 0 | 0 | 0 | 0 | 1 | ** |
| *Odontidium mesodon* (Kützing) Kützing | OMES | 0 | 0 | 0 | 0 | 1 | ** |
| *Pinnularia perirrorata* Krammer | PPRI | 0 | 0 | 0 | 0 | 1 | ** |
| *Planothidium frequentissimum* (Lange-Bertalot) Lange-Bertalot | PLFR | 0 | 0 | 0 | 0 | 1 | ** |
| *Sellaphora harderi* (Hustedt) J. Foets *et* C.E. Wetzel | SHRD | 0 | 0 | 0 | 0 | 1 | ** |
| *Tryblionella debilis* Arnott | TDEB | 0 | 0 | 0 | 0 | 1 | * |
| *Mayamaea fossalis* (Bock) Lange-Bertalot | MAFO | 0 | 0 | 0 | 1 | 0 | ** |
| *Sellaphora nana* (Hustedt) Lange-Bertalot, Cavacini, Tagliaventi *et* Alfinito | SENA | 0 | 0 | 0 | 1 | 0 | ** |
| *Adlafia minuscula* var*. minuscula* (Grunow) Lange-Bertalot | ASDMS | 0 | 0 | 0 | 1 | 1 | * |
| *Humidophila brekkaensis* (J.B. Petersen) Lowe, Kociolek, J.R. Johansen, Van de Vijver, Lange-Bertalot *et* Kopalová | HBRE | 0 | 0 | 0 | 1 | 1 | ** |
| *Microcostatus aerophilus* Stanek-Tarkowska, Noga, C.E. Wetzel *et* Ector | MAER | 0 | 0 | 0 | 1 | 1 | ** |
| *Nitzschia acidoclinata* Lange-Bertalot | NACD | 0 | 0 | 0 | 1 | 1 | ** |
| *Mayamaea* aff. *fossalis* (Krasske) Lange-Bertalot | MAFOa | 0 | 1 | 0 | 0 | 0 | ** |
| *Pinnularia microstauron* (Ehrenberg) Cleve var. *angusta* Krammer | PMIA | 0 | 1 | 0 | 0 | 0 | ** |
| *Navicula veneta* Kützing | NVEN | 0 | 1 | 0 | 0 | 1 | * |
| *Humidophila contenta* (Grunow) Lowe, Kociolek, J.R. Johansen, Van de Vijver, Lange-Bertalot *et* Kopalová | HCOT | 0 | 1 | 0 | 1 | 0 | ** |
| *Pinnularia molaris* (Grunow) Cleve | PMOL | 0 | 1 | 0 | 1 | 0 | * |
| *Nitzschia palea var. palea* (Kützing) W. Sm. | NPAL | 0 | 1 | 0 | 1 | 1 | ** |
| *Nitzschia perminuta* (Grunow) M. Peragallo | NIPM | 0 | 1 | 0 | 1 | 1 | ** |
| *Pinnularia subcapitata* f. *typica* J.B. Petersen | PSCT | 0 | 1 | 0 | 1 | 1 | ** |
| *Sellaphora atomoides* Wetzel *et* Van de Vijver | SEAT | 0 | 1 | 0 | 1 | 1 | ** |
| *Stauroneis parathermicola* Lange-Bertalot | SPTH | 0 | 1 | 0 | 1 | 1 | ** |
| *Mayamaea agrestis* (Hustedt) Lange-Bertalot | MAGR | 0 | 0 | 1 | 0 | 0 | ** |
| *Pinnularia subrupestris* Krammer | PSRU | 0 | 0 | 1 | 1 | 1 | * |
| *Pinnularia viridis* (Nitzsch) Ehrenberg var. *sudetica* (Hilse) Hustedt | PVSU | 0 | 0 | 1 | 1 | 1 | ** |
| *Sellaphora lundii* C.E. Wetzel, Barragán *et* Ector | SLUN | 0 | 1 | 1 | 0 | 0 | ** |
| *Hantzschia calcifuga* Reichardt et Lange-Bertalot | HCAL | 0 | 1 | 1 | 1 | 0 | ** |
| *Sellaphora subseminulum* (Hustedt) C.E. Wetzel | SSSE | 0 | 1 | 1 | 1 | 1 | ** |
| *Stauroneis thermicola* (J.B. Petersen) J.W.G. Lund | STHE | 0 | 1 | 1 | 1 | 1 | ** |
| *Luticola ventricosa* (Kützing) D.G. Mann | LVEN | 1 | 0 | 0 | 0 | 0 | ** |
| *Stauroneis* cf. *borichii* (J.B. Petersen) J.W.G. Lund | SBORv | 1 | 0 | 0 | 0 | 0 | ** |
| *Navicula cincta* (Ehrenberg) Ralfs var. *heufleri* Grunow f. *curta* | NCIH | 1 | 0 | 0 | 0 | 1 | * |
| *Stauroneis borichii* (J.B. Petersen) J.W.G. Lund | SBOR | 1 | 0 | 0 | 1 | 0 | ** |
| *Craticula minuscoloides* (Hustedt) Lange-Bertalot | CMNO | 1 | 1 | 1 | 0 | 0 | ** |
| *Mayamaea excelsa* (Krasske) Lange-Bertalot | MAEX | 1 | 1 | 1 | 0 | 0 | ** |
| *Surirella terricola* Lange-Bertalot *et* Alles | STER | 1 | 1 | 1 | 0 | 1 | ** |
| *Hantzschia abundans* Lange-Bertalot | HABU | 1 | 1 | 1 | 1 | 0 | ** |
| *Hantzschia amphioxys* (Ehrenberg) Grunow | HAMP | 1 | 1 | 1 | 1 | 0 | ** |
| *Luticola frequentissima* Levkov, Metzeltin *et* Pavlov | LFRQ | 1 | 1 | 1 | 1 | 0 | ** |
| *Mayamaea alcimonica* (Reichardt) Monnier *et* Ector | MALC | 1 | 1 | 1 | 1 | 0 | ** |
| *Mayamaea atomus* (Kützing) Lange-Bertalot | MAAT | 1 | 1 | 1 | 1 | 0 | ** |
| *Nitzschia pusilla* (Kützing) Grunow | NIPU | 1 | 1 | 1 | 1 | 0 | ** |
| *Pinnularia borealis* Ehrenberg | PBOR | 1 | 1 | 1 | 1 | 0 | ** |
